# The therapeutic potential of ayahuasca in depression, generalized anxiety, and substance use disorders: modulation of the depressive burden in a longitudinal study

**DOI:** 10.3389/fpsyt.2026.1726909

**Published:** 2026-01-29

**Authors:** Gláucio Silva Camargos, Geraldo Magela de Faria Júnior, Marcelo Lourenço, Gerardo Maria de Araújo Filho

**Affiliations:** 1 ¹ Department of Neurological Sciences, Psychiatry and Medical Psychology, Faculty of Medicine of São José do Rio Preto (FAMERP), São José do Rio Preto, São Paulo, Brazil; 2 Faculty of Medicine of São José do Rio Preto (FAMERP), São José do Rio Preto, São Paulo, Brazil; 3 UNI Shamanic Institute of São José do Rio Preto (IXUNI), São José do Rio Preto, São Paulo, Brazil

**Keywords:** ayahuasca, mental disorders, psychedelics, psychotherapy, resistant depression

## Abstract

**Introduction:**

This longitudinal observational study evaluated changes in depressive symptoms associated with ritualistic ayahuasca use in patients diagnosed with depressive disorders, as well as depressive burden in individuals with anxiety and substance use disorders.

**Methods:**

The research was conducted with 280 adults treated at clinics in the northwestern region of the state of São Paulo, under the jurisdiction of Regional Health Division XV (DRS XV), which covers a population of over 2 million inhabitants. Participants were assessed at six different time points over a six-month follow-up period after the ayahuasca intervention, with depressive symptoms measured using the Montgomery–Åsberg Depression Rating Scale (MADRS).

**Results:**

The results indicate a significant reduction in depression scores shortly after the intervention, with improvements sustained for up to 180 days, though individual responses varied. The analysis reveals that ayahuasca, when combined with psychotherapeutic support, can provide significant benefits in reducing symptoms of depression and anxiety, and also shows potential in the treatment of substance dependence.

**Conclusion:**

We concluded that the final variability in responses suggests that psychodynamic and clinical factors, such as the integration of the psychedelic experience and therapeutic support, play a crucial role in treatment effectiveness. This study contributes to the consolidation of psychedelic-assisted therapeutic protocols, highlighting the importance of continuous and personalized follow-up in the treatment of complex psychiatric conditions.

## 1 Introduction

**Figure 1:**
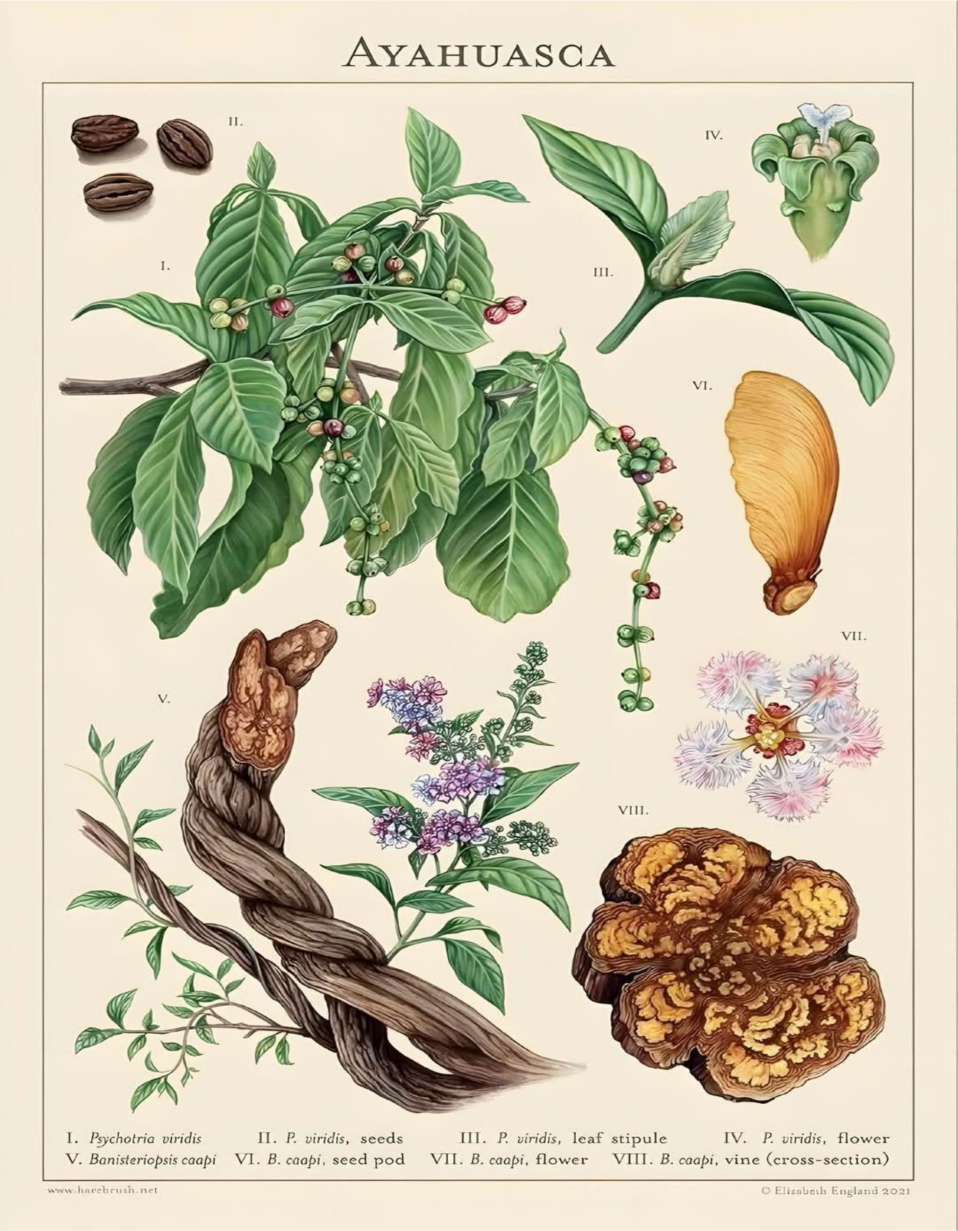
Ayahuasca, with botanical representation of the plants involved: *Banisteriopsis caapi* (Jagube) and *Psychotria viridis* (Mariri). Source: Elizabeth England, 2021 (3).

In recent years, the therapeutic use of psychedelic substances has garnered increasing interest within the scientific community, particularly in light of the urgent need for innovative approaches to treat mental disorders that are resistant to conventional therapies. Among these substances, ayahuasca stands out, a decoction traditionally used by Indigenous peoples of the Amazon in ritualistic and spiritual contexts. Prepared from the combination of two plants, *Banisteriopsis caapi* (Jagube) and *Psychotria viridis* (Mariri) (**Figure 1**), the brew contains β-carboline alkaloids (such as harmine) and dimethyltryptamine (DMT), which together produce intense psychic effects and significant alterations in states of consciousness (1, 2).

Recent clinical studies have shown that ayahuasca can promote rapid and sustained reductions in symptoms of depression and anxiety, even among patients with a history of resistance to conventional antidepressants (1, 4, 5). Furthermore, there is growing evidence that the mystical-type experiences elicited by psychedelic substances may be associated with profound processes of emotional, psychological, and spiritual restructuring, which could also benefit individuals with a history of substance use disorders (6–8). Additional findings indicate that ayahuasca may induce sustained improvements not only in depressive and anxious symptoms but also in addiction-related outcomes, including among treatment-resistant patients (8–10). Moreover, understanding the neurobiological mechanisms underlying the therapeutic response to ayahuasca (such as 5-HT2A receptor activation and MAO inhibition) continues to expand (11–13).

Despite these advances, significant gaps remain in the literature. Many studies involve small sample sizes, limited assessments, and a lack of medium- and long-term follow-up. Moreover, few studies have evaluated symptom progression using standardized instruments at multiple time points, which is essential for understanding the durability of ayahuasca’s therapeutic effects and its relationship with variables such as type of disorder and intensity of the subjective experience.

In light of this, the present study aimed to longitudinally assess the effects of ritualistic ayahuasca use on symptoms of depression, and modulation of the depressive burden in anxiety and substance dependence in a group of participants treated in a therapeutic setting in Brazil. The study focused on standardized psychometric measures collected at six distinct time points, seeking to contribute robust clinical evidence toward the development of psychedelic- assisted therapeutic protocols and to allow a detailed analysis of the temporal evolution of key clinical indicators.

## 2 Material and methods

This study employed a longitudinal observational design with repeated assessments over a 180-day follow-up period.

### 2.1 Ethical considerations

This study was approved by the Research Ethics Committee of the Faculty of Medicine of São José do Rio Preto (FAMERP) (CAAE 76957524.0.0000.5415). All participants signed an informed consent form (ICF) prior to enrollment.

### 2.2 Participants

The sample consisted of 280 adults diagnosed with depressive disorders, anxiety disorders, and/or substance use disorders. All participants were residents of the northwest region of the state of São Paulo (DRS XV – Regional Health Division XV), and received care through private clinics and practices within this same region. Participants were recruited using a convenience sampling strategy through clinical referral. Treating clinicians informed eligible patients about the study during routine appointments, and those who expressed interest received detailed information about the study procedures. As an inclusion criterion, all participants had formal psychiatric diagnoses established prior to study enrollment by licensed psychiatrists or physicians, based on standardized clinical criteria (DSM-5 or ICD-10) and documented in their medical records. Individuals who did not present a formal clinical diagnosis were excluded from the study. The informed consent form was reviewed and discussed with each participant individually.

Additional inclusion criteria were: (i) a documented clinical history of depression, anxiety, or substance use disorder; and (ii) completion of all psychometric assessments at every designated time point. Participants with active psychotic disorders or recent manic episodes were excluded from the study. No randomization or stratification procedures were employed. The informed consent form was reviewed and discussed individually with each participant.

### 2.3 Procedures

The intervention consisted of a ritual led by trained facilitators and included oral ingestion of ayahuasca, meditative practices, music, and therapeutic support. The ritual lasted five hours and was conducted by the UNI Shamanic Institute in São José do Rio Preto, SP, within a controlled environment, under professional supervision and appropriate guidance to ensure safety and containment. Participants attended three structured psychotherapeutic integration sessions delivered before and after the ayahuasca session. These sessions followed a psychodynamic framework and aimed to support emotional processing and integration of emergent material.

During the ceremony, each participant consumed 50 mL of ayahuasca during the first hour. After two hours, an additional 25 mL was administered. The ayahuasca used followed a 5:1 preparation ratio (five parts *Banisteriopsis caapi* to one part *Psychotria viridis*) representing a traditional concentration. The ayahuasca brew was prepared according to traditional procedures. However, its chemical composition (DMT and β-carboline concentrations) was not analytically quantified, which is inherent to the naturalistic design of the study. This preparation ratio reflects traditional practices rather than any experimental manipulation by the researchers.

Following the ritual, participants underwent psychodynamic psychotherapy sessions focused on integrating the material accessed during the experience and completing psychometric assessments. The structured psychodynamic integration sessions were carried out in an individual format and focused on emotional elaboration, meaning construction and the psychological integration of experiences. They lasted an average of 50 minutes and aimed to facilitate reflection on personal narratives, affective responses and symbolic contents emerging from the ritual experience. All integration sessions followed a structured psychodynamic format based on a predefined session outline to ensure consistency across participants. Therapists were trained psychologists with clinical experience in psychodynamic psychotherapy, and adherence to the framework was monitored through supervision meetings.

Participants were reassessed at six distinct time points: Pre-Ritual (baseline), Day 7, Day 14, Day 21, Day 90, and Day 180 after the ceremony. Assessments were conducted using standardized instruments administered by a trained technical team. All data were subsequently systematized in electronic spreadsheets.

### 2.4 Instruments

The Montgomery–Åsberg Depression Rating Scale (MADRS) was used as the primary tool for assessing depressive symptoms. Its use has also been validated in studies with populations affected by substance dependence, such as hospitalized alcoholic patients, in whom it showed adequate performance as a screening tool for depressive symptoms (14). In these contexts, the MADRS allows for quantification of the depressive burden associated with substance use or withdrawal, as well as monitoring of therapeutic response and the temporal evolution of symptoms. Considering that Generalized Anxiety Disorder (GAD) frequently co-occurs with significant depressive symptomatology, the MADRS becomes a suitable instrument for assessing overlapping affective dimensions. Its use is therefore methodologically justified as a primary or complementary measure of depressive outcomes in studies involving GAD and substance use disorders (15–17). Importantly, although the sample included individuals with anxiety and substance use disorders, the analyses focused exclusively on depressive symptom severity, as measured by the MADRS, thus capturing the extent of depressive symptomatology expressed within these comorbid clinical profiles (15–17). In addition, sociodemographic questionnaires and structured clinical interviews were conducted. Data were organized in spreadsheets and analyzed using both descriptive and inferential statistics. Longitudinal changes in MADRS scores were analyzed using repeated-measures ANOVA, with a significance level set at *p* < 0.05, an approach appropriate for modeling within-subject correlations across time. *Post-hoc* pairwise comparisons were performed with appropriate correction for multiple testing. Tukey’s *post hoc* test was also applied to identify specific time points with statistically significant differences. Effect sizes (partial eta squared for ANOVA and Cohen’s d for pairwise comparisons) were calculated to quantify the magnitude of observed changes over time. Outcome assessors were not blinded to the assessment time points, as blinding was not applicable in this naturalistic, non-randomized observational study.

Internal consistency metrics (e.g., Cronbach’s alpha) were not calculated for the current sample; however, the MADRS demonstrates well-established reliability in prior literature (15–17). Although standardized measures of subjective experience intensity (e.g., MEQ-30 and EDI) were collected, these data were not analyzed in the present manuscript and will be reported in a separate publication focusing specifically on experiential process variables.

## 3 Results

The sample included 280 participants (53% women, mean age 42.6 years) with an average 5.8-year illness duration. Major Depression (40.4%), Generalized Anxiety Disorder (25.2%), and Treatment-Resistant Depression (22%) were most common. Frequent medications included Sertraline/Clonazepam, Paroxetine/Clonazepam, and Fluoxetine/Zolpidem.

The analysis of MADRS scores over time revealed a clear trend of symptom reduction following the ayahuasca ritual, indicating a progressive decrease in depressive symptom severity among participants. This pattern is supported by the statistical findings:

**Table 1** indicates a decrease in mean MADRS scores throughout the study period, from 43.04 at baseline (pre-ritual) to 24.56 at Day 180, suggesting a consistent reduction in depressive symptoms or associated indicators. However, the increase in standard deviation from 4.48 at baseline to 10.52 at Day 180 reveals a growing variability in participants’ responses over time, with some individuals experiencing marked improvement while others showed minimal change or even worsening symptoms.

**Table 1:** Longitudinal variation in mean severity scores (MADRS) across different follow-up time points (pre-ritual to 180 days).

Time-Point	Mean	Standard deviation	Minimum	Median	Maximum M	IQR
Pre	43.04	4.48	30	43.0	52	6.00
D7	33.74	9.40	0	34.0	58	14.25
D14	21.99	10.28	7	22.0	50	17.00
D21	21.11	9.63	7	19.0	48	15.00
D90	22.52	9.90	7	23.0	51	14.00
D180	24.56	10.52	7	24.0	50	15.00

The median scores followed a similar downward trend, decreasing from 43.0 at baseline to 24.0 at Day 180, reinforcing the overall central tendency toward symptom improvement across the group. The interquartile range (IQR) also expanded over time, rising from 6.00 at baseline to 14.25 at Day 7, peaking at 17.00 on Day 14, and stabilizing around 15.00 by Day 180. This broader IQR reflects greater dispersion in scores, indicating increased heterogeneity in therapeutic outcomes.

Taken together, these findings suggest that although a general trend of clinical improvement was observed following the intervention, the magnitude and persistence of the observed reductions in depressive symptoms varied substantially among participants, highlighting the importance of considering individual differences and contextual variables in psychedelic-assisted treatment outcomes.

A significant decrease in mean scores was observed between the pre- ritual baseline (43.04) and subsequent time points, especially up to Day 14 (21.99). This improvement was maintained through Day 90 (22.52), with a slight upward trend at Day 180 (24.56), although scores remained substantially below the initial level. The standard deviation increased over time, indicating greater individual variability in treatment response.

A one-way repeated-measures ANOVA revealed a significant main effect of time on depressive symptoms (F(5, 140) = 89.25, p < 0.0001; partial η² = 0.761), with *post hoc* Tukey tests indicating significant reductions from baseline across all follow-up assessments. Consistent with this overall time effect, the effect-size estimates indicated substantial reductions in depressive symptom severity at all time points (**Table 2**), with the largest improvements observed on days 7 and 14 (large to very large effects), and slightly attenuated yet still significant reductions on days 90 and 180. These findings demonstrate that the magnitude of symptom improvement was not only statistically significant but also clinically meaningful.

**Table 2:** Effect sizes (Cohen’s d) and 95% confidence intervals for changes in MADRS scores across assessment time points.

Comparison	Cohen’s d (range)	95% CI (range)	Interpretation
Pre vs D7	0.81–1.06	0.68–1.21	Large
Pre vs D14	1.58–2.07	1.40–2.28	Very large
Pre vs D21	1.62–2.20	1.44–2.42	Very large
Pre vs D90	1.53–2.03	1.36–2.23	Very large
Pre vs D180	0.95–1.23	0.81–1.39	Large

Effect sizes (paired-samples Cohen’s d) represent the magnitude of change in MADRS depressive symptom scores from baseline to each follow-up time point. Interpretation followed conventional benchmarks (large ≥ 0.80; very large ≥ 1.30).

To specifically identify which time points differed significantly, Tukey’s HSD *post hoc* test was conducted. The results pinpointed the assessment periods that showed statistically significant differences in symptom scores.

Mean scores at the pre-intervention baseline were significantly higher than those observed at all subsequent time points (D7, D14, D21, D90, and D180), with *p* < 0.0001 for all comparisons. Statistically significant differences were also found between D14 and D7 (*p* < 0.0001), D21 and D7 (*p* < 0.0001), and D180 and D7 (*p* < 0.0001), suggesting a sharp decline in symptoms shortly after the intervention, particularly within the first two weeks.

Conversely, no significant differences were detected between D14, D21, and D90, nor between D90 and D180, indicating relative clinical stability in the medium to long term following ayahuasca administration. These findings support the hypothesis that the intervention produces a rapid and sustained improvement in depressive symptoms in a substantial proportion of the evaluated sample.

The graph above depicts the evolution of patients’ total scores over time, including data from multiple assessment moments (Pre-ritual, Day 7, Day 14, Day 21, Day 90, Day 180). The x-axis represents the total score, while the y-axis indicates frequency (number of participants attaining that score). Each color corresponds to a different assessment time point: yellow (pre-ritual), light salmon (D7), pink (D14), light cyan (D21), teal blue (D90), and dark blue (D180).

Histograms display the count of participants with specific scores. The smoothed curves (kernel density estimates - KDE) represent the theoretical distribution of data for each time point. The graph demonstrates a clear trend of score reduction. The yellow curve (pre-ritual) is shifted to the right, indicating higher scores (worse symptomatology). The pink curve (D14) also shows relatively elevated scores. Curves for D90 and D180 (blue hues) shift leftwards, suggesting a significant reduction in symptoms over time.

Scores show a gradual decrease following the ritual, with the peak improvement most evident at D90 and D180. The density curves become narrower and shift towards lower scores, reflecting symptom improvement and reduced variability.

Notably, some distributions are multimodal: at certain time points (e.g., D7 or D21), the curves exhibit more than one peak, indicating subgroups with differential response patterns among participants (e.g., responders versus non- responders). Additionally, there is persistence of the observed reductions in depressive symptoms, as the D180 curve remains well to the left of the baseline yellow line, indicating that the improvement observed following the intervention was still present six months later.

This demonstrates that symptom severity (measured by total MADRS scores) shows significant observed reductions shortly after the intervention, with improvement evident from the first days and the greatest reductions occurring between 90 and 180 days. The distribution of scores consistently shifts leftward over time, indicating sustained clinical improvement in most participants.

Initially, scores are more widely distributed and elevated, reflecting greater variability in symptom severity prior to treatment. Over time, especially after D7 and D14, a trend towards improvement emerges, with scores increasingly concentrated on the left, indicating symptom reduction. This trend continues through D21 and D90, with patients exhibiting progressively lower scores, reflecting treatment efficacy. By six months (D180), most patients display even lower scores, indicative of continued recovery or symptom stability. The trend lines highlight this progression, evidencing patients’ positive response to treatment over time.

The graph above reveals a general downward trend in scores during the initial weeks, followed by a slight rebound at the final assessment (Day 180), accompanied by an increase in the variability of scores over time. This pattern may indicate that, following the initial intervention, participants begin to diverge in their responses, with some maintaining higher scores while others sustain lower ones. Such analysis is useful for understanding the dynamics of the data across time and assessing the efficacy or association of the intervention or process.

**Table 3** with general statistics shows that, on average, scores are 27.83, indicating a decrease from the initial pre-ritual value of 43.04. The overall median of 23.50 suggests a right-skewed distribution, with some higher scores, particularly at Day 7, which may have pulled the mean upward. The overall standard deviation of 9.04 indicates substantial data dispersion, with scores varying considerably over time. The minimum value of 0, recorded at Day 7, shows extreme low scores in some cases, whereas the maximum of 58, also at Day 7, reflects that some participants still presented significantly elevated scores. The overall interquartile range (IQR) of 13.54 points to a considerable range among central data, consistent with the increased dispersion observed in the graph.

**Table 3:** Means, medians, variability, and overall range of symptom scores across all follow-up time points.

Item	Value
Overall Mean	27.83
Overall Median	23.50
Overall Standard Deviation	9.04
Overall Minimum	0.00
Overall Maximum	58.00
Overall Interquartile Range (IQR)	13.54

## 4 Discussion

This study aimed to investigate changes in depressive symptoms associated with ayahuasca use through longitudinal psychometric evaluation. The results indicate reductions in MADRS scores, with improvements evident soon after the ritual and partial sustainment of these reductions up to 180 days. The observed variability in therapeutic response may be related to psychodynamic, clinical, and contextual factors that were not directly measured in the present study and should be further explored in future research (1, 13–18).

The present findings demonstrate reductions in depressive symptoms over time, with the largest improvements emerging within the first two weeks following the intervention. These early reductions align with previous reports describing rapid symptomatic improvement in similar naturalistic contexts (19–30). Although the magnitude of the reductions gradually diminished at later follow-up points, improvements remained substantial across the full 180-day period (6 months), indicating a sustained pattern of symptomatic relief. As shown in **Table 2**, overall, the trajectory of the results suggests statistically significant and clinically relevant reductions in the severity of depressive symptoms over the observation period.

Global literature has demonstrated positive effects of ayahuasca in treatment-resistant depression and anxiety (1, 20–22), corroborating the findings of this study. However, the heterogeneous participant responses over time represent a critical aspect seldom deeply explored (23–26).

**Table 1** reveals a pronounced decline in mean MADRS scores between the pre-ritual baseline (43.04) and Day 14 (21.99), remaining reduced through Day 90 (22.52), with a slight increase at Day 180 (24.56). Meanwhile, the standard deviation progressively increased from 4.48 (pre) to 10.52 (D180), indicating growing dispersion in responses. This demonstrates that, although the mean improvement is significant, it occurs unevenly across patients, with some showing rapid and intense responses, others modest improvement, or even transient worsening (27–31).

**Figure 2:**
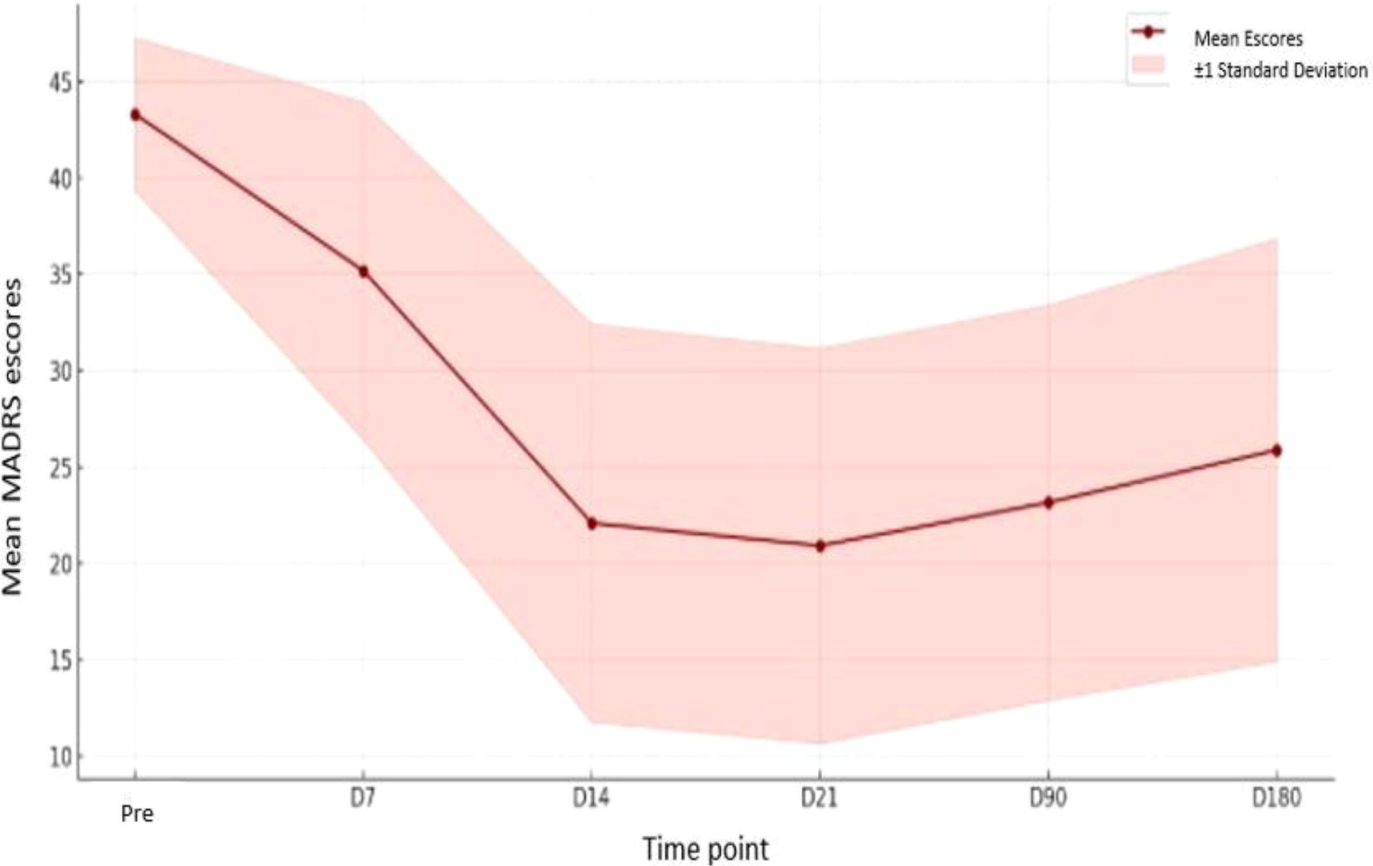
Longitudinal changes in mean MADRS scores over six months following the ayahuasca intervention. The shaded area represents ±1 standard deviation, highlighting interindividual variability across assessment points.

This dynamic is reinforced by **Figure 2**, illustrating the average trajectory of scores and the widening range of variation over time. The descending curve in the first 14 days reflects a therapeutic pattern commonly seen in psychedelic studies (32–34), followed by a clinical stabilization phase, demonstrating consolidation of psychic gains with appropriate integration (35–38). This phenomenon aligns with psilocybin literature (38), which also shows that long-term effects depend on variables such as ongoing therapeutic support and integration of emergent content (19).

**Figure 3:**
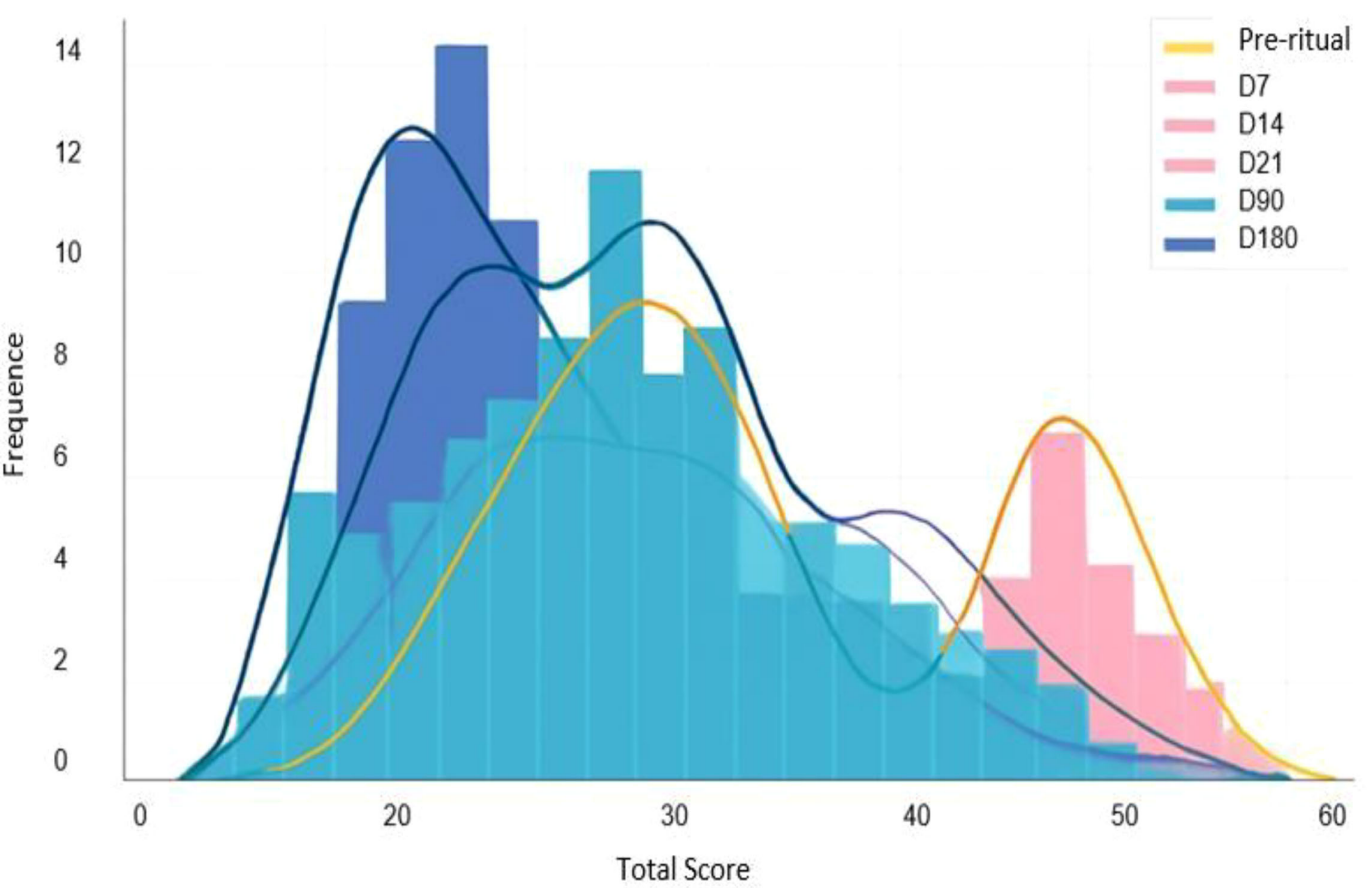
Distribution of total scores over time at assessment points: pre-intervention and post-intervention. The density curves illustrate a gradual and sustained reduction in scores, with a leftward shift of the distributions at subsequent time points, indicating progressive clinical improvement. Histograms represent the frequency of participants obtaining specific scores at each temporal point.

**Figure 3** presents the distribution of individual scores per time point. Early days (D7 and D14) show a concentration of lower scores with reduced dispersion. However, from D90 and especially D180, scores disperse again, with patients at distinct extremes. This is consistent with investigations on other psychedelics such as psilocybin (38) and LSD (23), which highlight that long-term efficacy depends on continuous therapeutic support and integration of psychedelic content (1, 39, 40).

**Figure 4:**
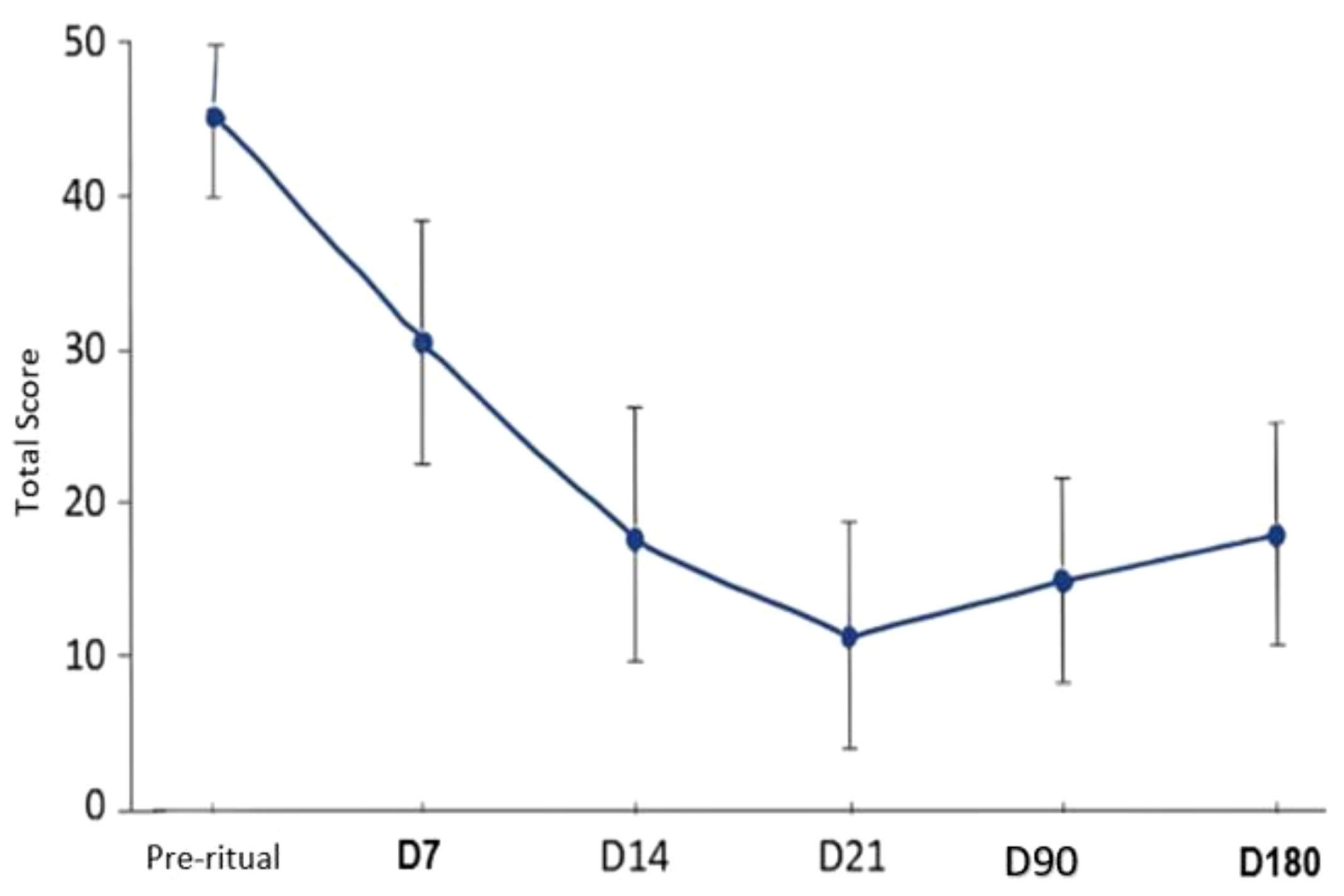
Mean MADRS total scores across assessment points from baseline to six-month follow-up. Error bars represent ±1 standard deviation, illustrating the central tendency and dispersion of depressive symptom severity over time.

The individual variability over time, as shown in **Figure 4**, confirms this clinical oscillation, demonstrating how the initial observed reductions tend to stabilize or diverge after the first three months Recent studies suggest that these variations may be amplified by psychodynamic factors, such as how patients integrate psychedelic experiences during therapy (19, 41–45).

**Table 3**, compiling general statistics (mean: 27.83; median: 23.5; standard deviation: 9.04), summarizes the global behavior of scores. The difference between mean and median suggests right skewness (presence of elevated scores even post-intervention) warranting personalized clinical attention (27, 42, 44, 46).

### 4.1 *Post Hoc* Tukey HSD test results

Detailed comparisons between time points are shown in **Table 4**: the most substantial difference was between Pre and Day 14 (21.22 points; *p* < 0.0001), followed by significant differences between Day 7 and Day 14, and Day 7 and Day 180, demonstrating that the largest drops occurred within the first weeks. Conversely, comparisons among Day 14, Day 21, and Day 90 were not significant, suggesting maintenance of the observed reductions after the initial improvement peak (25, 44–48).

**Table 4:** Multiple comparisons between assessment time points: Tukey’s HSD *post hoc* test results for differences in symptom scores.

Comparison	Mean difference	p-value	Significant difference
Pre vs D7	8.14	< 0.0001	Yes
Pre vs D14	21.22	< 0.0001	Yes
Pre vs D21	22.40	< 0.0001	Yes
Pre vs D90	20.15	< 0.0001	Yes
Pre vs D180	17.43	< 0.0001	Yes
D7 vs D14	-13.09	< 0.0001	Yes
D7 vs D21	-14.26	< 0.0001	Yes
D7 vs D90	-12.02	< 0.0001	Yes
D7 vs D180	-9.30	< 0.0001	Yes
D14 vs D21	-1.18	0.9509	No
D14 vs D90	1.07	0.9678	No
D14 vs D180	3.79	0.0530	No
D21 vs D90	2.24	0.5458	No
D21 vs D180	-4.97	0.0030	No
D90 vs D180	-2.72	0.3210	No

These results support findings by Palhano-Fontes et al. (8), who reported a rapid and lasting antidepressant effect after a single ayahuasca dose. The present data, with a 180-day follow-up, expand knowledge on the duration and fluctuation of these observed reductions in depressive symptoms over time. The progressive increase in variance over time suggests heterogeneous long-term trajectories, which is consistent with real-world clinical populations (5, 48–50).

### 4.2 Importance of psychotherapeutic integration

Clinical stability between Day 14 and Day 90, evidenced by multiple non- significant comparisons (**Table 4**) and the shape of the curve in **Figure 2**, points to the efficacy of the psychodynamic integration model used. Recent studies (27, 28, 50) highlight that ayahuasca experiences, if not symbolically processed, may lose therapeutic value or generate contradictory effects (6, 51).

In this study, clinical support before, during, and after the ritual was structured within psychodynamic frameworks, which may have supported the consolidation of the observed reductions in depressive symptoms, aligning with international evidence (23, 27, 52–54). This is consistent with psychodynamic integration therapy approaches described by Mertens et al. (24) and Olivieri et al. (55), suggesting that combining psychodynamic therapies with psychedelics may facilitate internal conflict resolution and promote more effective psychological growth.

### 4.3 Association of psychiatric comorbidities

Most patients presented severe and chronic diagnoses, such as treatment- resistant depression (22%), with high prevalence of anxiety comorbidities and ongoing psychopharmacological treatments (e.g., Sertraline, Paroxetine, Clonazepam) (**Table 5**). This clinical profile is particularly challenging and less responsive to conventional treatments (5, 8, 27, 53). Nevertheless, the data indicate that these patients, even under pharmacotherapy, responded positively to ayahuasca, corroborating studies by Bouso et al. (27) and Carhart-Harris et al. (32) on ayahuasca’s applicability in difficult-to-treat clinical contexts (50, 56–58).

**Table 5:** Sociodemographic profile, clinical history, and predominant pharmacological treatments among study participants (*n* = 280).

Indicator	Value
Number of participants	280
Mean age (years)	42.6 ± 11.3 (range: 20–64)
Female participants	150 participants (53%)
Male participants	130 participants (47%)
Mean duration since diagnosis (years)	5.8 ± 3.2
Most common diagnosis 1	Major Depression (n=144; 40,4%)
Most common diagnosis 2	Generalized Anxiety Disorder (n=71; 25,2%)
Most common diagnosis 3	Treatment-Resistant Depression (n=62; 22%)
Most frequent medications	Sertraline/Clonazepam (22,7%)
	Paroxetine/Clonazepam (21,3%)
	Fluoxetine/Zolpidem (20,9%)

Values are presented as mean ± standard deviation (SD) or frequency (%), as appropriate.

Although no conclusions can be drawn regarding changes in anxiety or substance-related symptoms, the analyses focused exclusively on depressive symptom severity (MADRS), capturing the depressive burden present in individuals with these comorbid conditions (15–17).

From a psychoneuroimmunological perspective, the interaction among nervous, immune, and endocrine systems is crucial to understanding ayahuasca’s therapeutic efficacy. As a psychedelic, ayahuasca may induce significant changes in gene expression and regulation of neurotrophic proteins, as well as modulate stress response systems (22, 59–61). These changes can alter brain neurochemistry, enhancing neuronal plasticity and recovery of regions involved in emotion and memory, which may explain observed improvements in patients with resistant depressive symptoms.

### 4.4 Clinical implications

Evidence suggests that ayahuasca, when integrated into psychotherapeutic models, is associated with observed reductions in depressive symptoms among individuals with treatment-resistant depression, particularly those with multiple comorbidities. **Tables 5**, **1**, **4** and **Figures 1**-**4** support this potential and emphasize the need for ongoing monitoring and individualized adjustments according to clinical response (22–24).

Ethical, safe, and evidence-based use of ayahuasca requires trained professionals able to manage the complexity of psychedelic experience and integrate cultural, spiritual, and psychoemotional aspects (62). The model described here may serve as a reference for future therapeutic protocols but must be adapted to each clinical context (58–62).

### 4.5 Limitations

This study has limitations that should be considered when interpreting the results. First, the chemical composition of ayahuasca was not analytically quantified, which limits pharmacological characterization and reflects the inherent restrictions of naturalistic studies. Second, although the use of psychiatric medication was documented and monitored during follow-up, these variables were not included in the current analyses as they will be examined in a separate publication focused specifically on pharmacological effects. No additional psychedelic experiences were administered or reported during the follow-up period. Third, as this was a naturalistic observational study without a control group or randomization, blinding of the raters was not feasible. Although validated measures of the intensity of the psychedelic experience were collected, these variables were not analyzed in this study and will be explored in a dedicated future analysis.

Furthermore, several contextual and clinical factors, such as the availability of social support and the characteristics of the ritualistic environment, may have contributed to the observed reductions in depressive symptoms. The absence of physiological measures (e.g., neuroimaging or biomarkers) also restricts the interpretation of underlying neurobiological mechanisms. Furthermore, because the sample was regionally and culturally homogeneous, generalization to broader populations is limited (32, 36). Finally, the observational nature of this study precludes causal inferences.

Future research should incorporate biochemical characterization of ayahuasca, multimodal physiological assessments, measures of experience intensity, and multicenter designs to improve methodological rigor and elucidate the mechanisms underlying the clinical alterations associated with ayahuasca.

## 5 Conclusion

This study contributes to the understanding of the therapeutic use of ayahuasca in the treatment of depressive symptoms, highlighting the ritual context as a psychotherapeutic intervention associated with significant reductions in symptom severity. The findings highlight the ritual’s efficacy as a psychotherapeutic intervention, demonstrating substantial symptom reduction and modulation of the depressive burden in a longitudinal study up to 180 days post-administration, with heterogeneous response among participants, reinforcing the importance of a personalized, integrative therapeutic approach. The observation of substantial initial improvement followed by gradual stabilization suggests ayahuasca may promote durable psychological gains when accompanied by adequate psychotherapeutic strategies. Final variability in individual responses is important to consider, with some patients showing rapid and intense improvement, while others experience slower progression or transient worsening.

These findings underscore the need for careful analysis of psychodynamic, clinical, and contextual factors that may influence the trajectory of depressive symptom changes, including the provision of psychotherapeutic support before, during, and after the intervention. Additionally, the data highlight psychiatric comorbidities as key determinants of treatment response, corroborating previous research indicating ayahuasca’s efficacy in complex, treatment-resistant clinical cases.

Psychotherapeutic integration based on psychodynamic models may have contributed to sustaining the observed reductions, indicating that continuous therapeutic follow-up may be associated with greater stability of outcomes in ayahuasca-assisted settings.

Clinically, these results indicate that ayahuasca, combined with an integrative psychotherapeutic approach, may represent a valid alternative in treatment-resistant depression, particularly in patients with multiple psychiatric comorbidities. However, it is essential that healthcare professionals are prepared to manage the complexity of the psychedelic experience and provide continuous support enabling true integration of the therapeutic process.

Development of clinical protocols based on adaptive psychotherapeutic models will be essential for safe and effective implementation of ayahuasca as a psychotherapeutic treatment in the future.

## Data Availability

The raw data supporting the conclusions of this article will be made available by the authors, without undue reservation.
